# Supplementary material for: Outcomes and clinical implications of intranasal insulin on cognition in humans: A systematic review and meta-analysis
Source: PLoS One. 2023 Jun 28;18(6):e0286887. doi: 10.1371/journal.pone.0286887 (PMC10306194; doi:10.1371/journal.pone.0286887)

Supplementary Table 1. Database Search String

Database: Ovid MEDLINE: Epub Ahead of Print, In-Process & Other Non-Indexed Citations, Ovid MEDLINE® Daily and Ovid MEDLINE® <1946-Present>

#	Query	Results from 10 Jun 2021
1	executive function/ or executive function.mp.	27,215
2	learning/ or learning.mp.	435,240
3	memory/ or memory.mp.	299,171
4	problem solving/ or problem solving.mp.	38,194
5	decision making/ or decision making.mp.	232,383
6	cognition.mp.	216,277
7	cognitive func*.mp.	68,198
8	Attention/ or attention.mp.	497,457
9	(verbal memory or visuospatial memory or executive function or problem solving or working memory or declarative memory or visual memory or visual learning).tw,kf.	76,383
10	working memory.mp.	34,382
11	1 or 2 or 3 or 4 or 5 or 6 or 7 or 8 or 9 or 10	1,461,168
12	insulin, long-acting/ or insulin, short-acting/	3,020
13	insulin.mp.	431,116
14	Administration, Intranasal/ or intranasal administration.mp. or intranasal insulin.mp.	16,835
15	(intranasal insulin or nasal insulin or nasal spray or intranasal administration).tw,kf.	6,856
16	12 or 13 or 14 or 15	448,920
17	11 and 16	11,514
18	Humans/ or humans.mp. or humans.tw,kf.	19,488,275
19	17 and 18	7,475
20	randomized controlled trial.mp. or exp Randomized Controlled Trial/ or RCT.mp. or (randomized controlled trial or RCT).tw,kf.	583,549
21	19 and 20	646

Supplementary Table 2: Characteristics of Included Studies

Study	Intervention	Duration of Tx	Total Dose (IU)	Patient Sample	*N*	% Male	Age (years)	Education	Baseline BMI	Serum Insulin Levels before/after INI Administration	INI Side Effects	Cognitive Measures	Outcomes of Assessments
**Benedict et al. 2004**	Placebo or 4 x 40 IU/day RH-I	8 weeks	160	Healthy participants	*N* = 38<br>INS *N* = 19<br>PBO *N* = 19	63.16	INS: 25.26 (1.21)<br>PBO: 25.63 (1.25)	NR	INS: 22.6 (0.3)<br>PBO: 22.7 (0.4)	- No difference in plasma INS and glucose concentrations were observed between the INS and PBO groups<br>- ↓ serum cortisol observed in INS group compared to PBO group	No side effects reported.	- Word List<br>- Wordstem Priming Task<br>- Stroop Test	- INI improved delayed recall of words on the word list, but had no effect on immediate recall. Memory was particularly improved for emotional and neutral words on the list<br>- INS and PBO groups performed similarly on the wordstem priming task and Stroop test (including all three subtests)
**Benedict et al. 2007**	Placebo, 4 x 40 IU/day RH-I, or 4 x 40 IU/day ASP-I	8 weeks	160	Healthy participants	*N* = 36<br>RH-I *N* = 12<br>ASP-I *N* = 12<br>PBO *N* = 12	100	INS:<br>RH-I: 24.92 (1.63)<br>ASP-I: 24.42 (1.33)<br>PBO:<br>26.25 (1.66)	NR	< 25	- No difference in plasma glucose and serum INS levels following both acute and subchronic INI treatment	No side effects reported.	- Immediate and Delayed Word List Recall<br>- Declarative Memory Test	- INI treatment improved delayed recall of words (ASP-I had a stronger effect than RH-I, although both enhanced delayed recall compared to PBO)<br>- INI did not improve perofrmance on immediate recall
**Benedict et al. 2008**	Placebo and 1 x 160 IU RH-I	Acute	160	Healthy participants	*N* = 32	22.37	22.44 (0.63)	NR	NR	- ↓ plasma glucose and c-peptide levels following INI; these changes were not related to food intake and memory performance outcomes<br>- No difference in concentrations of circulating INS, cortisol, leptin, and adiponectin were observed after INI administration	NR	- Digit Span<br>- Two-dimensional (2-D)-object Location Task<br>- Mirror tracing	- Acute INI improved 2-D-object location task and verbal working memory performance in women but not in men<br>- Acute INI did not improve performance on procedural mirror tracing task in both genders
**Feld et al. 2016**	Placebo or 1 x 160 IU Insulin Actrapid	Acute	160	Healthy participants	*N* = 32	50	Range: 18-30	NR	NR	- ↑ in INS concentration immediately after INI administration compared to PBO group; however the effect was transient	NR	- Word-pair Interference Paradigm- Finger	- Acute INI had no effect on the retrieval of declarative and procedural memory traces acquired before sleep
**Gwizdala et al. 2021**	Placebo or 0 to 120 IU single dose ASP-I	Acute	0 - 120	Non-diabetic, fasted college-aged adults	*N = 116*<br>*0 IU N = 18*<br>*20 IU N = 16*<br>*40 IU N = 17*<br>*60 IU N = 15*<br>*80 IU N = 15*<br>*100 IU N = 18*<br>*120 IU N = 17*	37.93	20.7 (2.5)	13.9 (2.2) years	NR	NR	Side effects reported:<br>- Lightheaded/dizziness<br>- Blurred vision<br>- Unusual fatigue<br>- Jittery<br>- Headache<br>- Burning/tingling in nose<br>- Tingling in mouth<br>- Watering/tearing of eyes<br>- Nose bleed/runny nose<br>- Swelling/soreness of nose	- Inhibitory Control Task<br>- Rapid Visual Information Processing Task	- INI had no effect on response accuracy, regardless of dose administered, during both the inhibitory control and sustained attention tasks
**Krug et al. 2010**	Placebo and 1 x 160 IU RH-I	Acute	160	Healthy postmenopausal women	*N* = 14	0	57.61 (1.14)	NR	23.71 (0.6)	- Slight ↓ in plasma glucose concentrations after INI administration; effects were not statistically significant	NR	- Digit Span<br>- 2-D-Object Location Task	- Acute INI improved verbal working memory in women of all ages<br>- Acute INI did not improve performance 2-D-object location task in women
**Hamidovic et al. 2018**	Placebo or 1 x 60 IU RH-I	Acute	60	Abstinent smokers	*N* = 50<br>INS *N* = 26<br>PBO N = 24	PBO 66.67<br>INS: 80.77	INS:<br>36.5 (11.82)<br>PBO:<br>38.3 (13.33)	INS:<br>12th grade or less: 11.54%<br>High school graduate: 23.08%<br>Some college/AA degree: 53.85%<br>College graduate: 7.69%<br>Graduate school degree: 3.85%<br>PBO:<br>12th grade or less: 0.00%<br>High school graduate: 29.17%<br>Some college/AA degree: 50.00%<br>College graduate: 20.83%<br>Graduate school degree: 0.00%	NR	NR	NR	- CVLT-II	- INI did not improve learning over the five verbal learning trials<br>- INI did not improve either short- or long-delay recall in either study
**Claxton et al. 2015**	Placebo, 2 x 10 IU/day Det or 2 x 20 IU/day Det	3 weeks	20 or 40	MCI or mild to moderate AD	*N* = 60<br>20 IU *N* = 21<br>40 IU *N* = 19<br>PBO *N* = 20	PBO: 65<br>20 IU: 61.9<br>40 IU: 57.9	INS:<br>20 IU: 71.76 (1.88)<br>40 IU: 72.74 (1.93)<br>PBO:<br>71.45 (1.87)	INS:<br>20 IU: 15.95 (0.60) years<br>40 IU: 15.58 (0.55) years<br>PBO:<br>16.70 (0.43) years	NR (Supplementary Table 4 reports cholesterol summary for all subjects)	- No differences in mean fasting plasma glucose across all treatment groups (20 IU, 40 IU, and PBO)	Total cases of adverse events: 29- Light-heade	- Immediate and Delayed Story Recall<br>- Immediate and Delayed Word List Recall<br>- Dot Counting N-back<br>- BVRT<br>- Stroop Test	- INI improved performance for verbal and visuospatial working memory in the 40 IU group compared to PBO<br>- INI did not improve verbal memory and executive function after three weeks of treatment
**Claxton et al. 2013**	Placebo, 20 IU/day INI, or 40 IU/day RH-I	16 weeks	20 or 40	Older adults with amnestic MCI or mild to moderate AD	*N* = 104<br>20 IU *N* = 36<br>40 IU *N* = 38<br>PBO *N* = 30	56.73	INS:<br>20 IU: 72.5 (7.6)<br>40 IU: 69.75 (8.9)<br>PBO:<br>74.6 (10.05)	INS:<br>20 IU: 15.45 (3.25) years<br>40 IU: 16.25 (2.8) years<br>PBO:<br>15.1 (3.05) years	INS:<br>20 IU: 26.35 (3.65)<br>40 IU: 26.85 (4.95)<br>PBO:<br>27.25 (4.0)	- No differences in mean fasting plasma glucose across all treatment groups (20 IU, 40 IU, and PBO)	No reports of treatment related serious adverse events, minor reports of mild rhinitis and infrequent nose bleeds.	- Delayed Story Recall<br>- ADAS-Cog	- INI improved delayed story recall in the 20 IU group after four months of INI treatment compared to PBO, this effect was not found for the 40 IU group<br>- Significant sex interaction observed for delayed story recall, men in the 40 IU group improved after four months of INI treatment, but not women<br>- INI did not improve ADAS-Cog scores in both genders

**Craft et al. 2012**	Placebo, 2 x 10 IU/day RH-I, or 2 x 20 IU/day RH-I	16 weeks	20 or 40	Adults with amnestic MCI or mild to moderate AD	*N* = 104<br>20 IU *N* = 36<br>40 IU *N* = 38<br>PBO *N* = 30	PBO: 56.7<br>20 IU: 61.1<br>40 IU: 52.6	INS:<br>20 IU: 72.8 (1.5)<br>40 IU: 69.9 (1.4)<br>PBO:<br>74.9 (1.6)	INS:<br>20 IU: 15.5 (0.5) years<br>20 IU: 16.2 (0.5) years<br>PBO: 15.3 (0.6) years	INS:<br>20 IU: 26.7 (0.8)<br>40 IU: 26.9 (0.7)<br>PBO:<br>27.4 (0.8)	NR	Total cases of adverse events: 133<br>- Light-headedness/dizziness<br>- Headache (not related to lumbar puncture)<br>- Nose bleed<br>- Rhinitis<br>- Upper respiratory tract infection<br>- Fall<br>- Rash<br>- Other	- Delayed story recall<br>- DSRS<br>- ADAS-Cog	- INI improved delayed story recall in the 20 IU group compared to PBO group, this effect was not found for the 40 IU group<br>- INI did not improve DSRS scores for both INS groups compared to PBO group<br>- Both INS groups also showed less decline in ADAS-Cog scores compared to PBO group.
**Craft et al. 2017**	Placebo, 2 x 20 IU/day Det, or 2 x 20 IU/day RH-I	16 weeks	40	Adults with MCI or mild to moderate AD	*N* = 36<br>40 IU Det *N* = 12<br>40 IU RH-I *N* = 12<br>PBO *N* = 12	47.22	INS:<br>40 IU RI: 70.5 (9.1)<br>40 IU Det: 67.3 (7.8)<br>PBO:<br>68.4 (8.9)	INS:<br>40 IU RH-I: 15.6 (2.8) years<br>40 IU Det: 14.8 (2.4) years<br>PBO:<br>16.5 (2.0) years	INS:<br>RH-I: 28.8 (6.1)<br>Det: 29.4 (6.6)<br>PBO:<br>26.7 (3.3)	NR	No side effects reported.	- Delayed story recall<br>- Delayed selective reminding test recall<br>- ADAS-Cog<br>- DSRS	- RH-I improved memory after two and four months compared to the PBO group, no effects were observed in the Det group<br>- INI did not improve ADAS-Cog and DSRS scores
**Craft et al. 2020**	Placebo or 40 IU/day RH-I	52 weeks	40	Amnestic MCI and AD	*N* = 240<br>INS *N* = 121 PBO *N* = 119	51.3	INS:<br>70.5 (7.4)<br>PBO:<br>71.1 (6.8)	INS:<br>16.1 (2.8) years<br>PBO:<br>16.3 (2.9) years	NR	- No difference in blood glucose levels were observed after INI administration	List of adverse events reported in Supplementary Table 4.	- ADAS-Cog	- INI did not improve ADAS-Cog scores after 12 months of INI treatment
**Kellar et al. 2021**	Placebo or 2 x 20 IU/day RH-I	52 weeks	40	Adults with MCI or AD	*N* = 40<br>INS *N* = 20<br>PBO *N* = 20	62.5	INS:<br>71.69 (8.25)<br>PBO:<br>70.88 (5.69)	INS:<br>16.05 (2.87) years<br>PBO:<br>17.2 (2.48) years	NR	NR	NR	- ADAS-Cog	- INS group showed a significant correlation between change in ADAS-Cog scores and white matter hyperintensity volume change in the corpus callosum, and trending correlations for the deep white matter and temporal regions
**Mustapic et al. 2019**	Placebo, 20 IU/day RH-I, or 40/day IU RH-I	16 weeks	20 or 40	Amnestic MCI or probable AD	*N* = 91<br>20 IU *N* = 33<br>40 IU *N* = 32.<br>PBO *N* = 26	38.46	INS:<br>20 IU 70.9 (6.83)<br>40 IU 69.6 (9.13)	NR	NR	NR	No side effects reported.	- ADAS-Cog	- INI treatment of 20 IU showed changes in cognition that were positively correlated with changes in extracellular vesicles biomarkers<br>- No significant changes in cognition observed in both PBO and 40 IU treatment groups
**Reger et al. 2006**	Placebo, 20 IU RH-I, or 40 IU RH-I	Acute	20 or 40	Amnestic MCI and early AD	N = 61<br>AD/MCI: *N* = 26<br>HC: *N* = 35	45.9	AD/MCI: 76.7<br>HC: 74.2	AD/MCI: 14.2 (3.2)<br>HC: 15.4 (2.2)	HC:<br>ε4-: 25.6±3.0<br>ε4+: 25.9±4.3<br>MCI:<br>ε4-: 24.0±3.8<br>ε4+: 24.3±2.3<br>AD:<br>ε4-: 25.2±1.7<br>ε4+: 25.4±2.8	- No difference in plasma INS and glucose levels were observed in any of the diagnostic groups following INI administration	- One subject experienced a minor nosebleed that lasted for severalminutes the evening after receiving 40 IU insulin.<br><br>- A second subject experienced nose soreness for about 24 h that beganwhen the needleless syringe was inserted into the nasal cavity.	- Immediate and Delayed Story Recall<br>- Selective Reminding Word List Task<br>- Self-Ordered Pointing Task<br>- Stroop Test<br>- Visual Search	- INI facilitated recall on two measures of verbal memory in memory-impairedε4-adults<br>- Effects of INI were stronger for memory-impairedε4- subjects than for memory-impairedε4+ subjects and normal adults.<br>- Memory-impaired ε4+ subjects showed poorer recall following insulin administration on onetest of memory.
**Reger et al. 2008a**	Placebo, 10 IU, 20 IU, 40 IU, or 60 IU RH-I	Acute	10, 20, 40, or 60	Amnestic MCI and AD	N = 92<br>AD/MCI: n = 33<br>HC: 59	N/A	AD/MCI: 76.6<br>HC: 73.2	AD/MCI: 14.6 (0.7)<br>HC: 14.9 (0.6)	HC:<br>ε4-: 26.1±0.6<br>ε4+: 25.6±1.2<br>MCI:<br>ε4-: 26.4±1.2<br>ε4+: 26.5±0.8	- No difference in blood glucose and INS levels after INI administration in memory-impaired and normal subjects	NR	- Immediate and Delayed Story Recall<br>- HVLT<br>- Stroop Teset<br>- Self-Ordered Pointing Task<br><br>- DSST	- INI administration facilitated recall for adults with memory impariment for immediate and delayed recall as well as list learning<br>- Dose-dependent effect of INI on memory performance, with performance generally peaking at 20 IU
**Reger et al. 2008b**	Placebo or 2 x 20 IU/day RH-I	3 weeks	40	Amnestic MCI and early AD	*N* = 25<br>INS *N* =13<br>PBO N = 12	N/A	INS:<br>77.1 (1.6)<br>PBO:<br>79.3 (1.7)	INS:<br>14.9 (0.8) years<br>PBO:<br>15.5 (0.9) years	INS:<br>26.9 (1.2)<br>PBO:<br>26.0 (1.3)	- No difference in blood glucose and insulin levels after INI administration for all conditions	NR	- Immediate and Delayed Story Recall<br>- Stroop Test	- INI-treated groups retained more verbal information after a delay period and improved selective attention compared to the PBO-treated groups
**Rosenbloom et al. 2014**	Placebo, 20 IU GLU	1 week	20	Mild to moderate AD	*N* = 12<br>GLU *N* = 6<br>PBO *N* = 6	75	72	NR	NR	- ↓ serum INS levels temporarily follow INI treatment (not maintained in subsequent visits)	No side effects reported.	- RBANS<br>- WAIS-IV Digit Span Subtest<br>- TMT-A<br>- TMT-B<br>- BNT	- INI-treated group showed significantly higher scores on the RBANS line orientation task and improved performance on trails B test compared to the PBO group<br>- No other significant differences between PBO and INI groups in terms of learning, memory, executive functioning, language, and visuospatial function
**Rosenbloom et al. 2021**	Placebo or 2 x 20 IU/day GLU	32 weeks	40	Amnestic MCI and early AD	*N* = 35<br>GLU *N* = 19<br>PBO *N* = 16	57.1	68.4 (8.1)	Masters/PHD: 26.3%<br>Bachelors/associate: 47.4%<br>High school/equivalent: 26.3%	INS:<br>25.0 (4.4)<br>PBO:<br>24.5 (3.6)	- No difference in blood glucose or rate of hypoglycemia were observed between the treatment and PBO groups	- The number of adverse events per person was similar between treatment groups<br><br>- Subjects receiving intranasal GLU had higher rates of nasal irritation and respiratory symptoms compared to PBO<br><br>- One serious adverse event occurred in the insulin group and was classified as moderate light-headedness or dizziness and was probably not related to drug	- ADAS-Cog<br>- CDR Global Score<br>- FAQ<br>- Digit Span<br>- TMT-A<br>- TMT-B<br>- COWAT<br>- WMS-IV Logical Memory	- INI had no effect on ADAS-Cog scores and CDR global scores after three and six months of treatment compared to PBO group<br>- Function as measured by the FAQ generally worsened over time in both groups, with there being no significant difference between groups<br>- When comparing individual cognitive modalities, there was no significant impact of INI on verbal recall/retrieval, digit span, trail making, or verbal fluency (as measured by the COWAT)
**Stein et al. 2011**	High dose D/placebo or 4 x 60 IU/day RH-I	8 weeks	240	Mild to moderate AD	*N* = 32<br>INI *N* = 16<br>PBO *N* = 16	46.88	77.5	NR	24.5 (22-26.5)	NR	NR	- ADAS-Cog<br>- WMS-Revised Logical Memory Subtest for Immediate and Delayed Recall	- INI treatment improved ADAS-Cog, but not WMS-RLM scores, in patients with mild to moderate Alzheimer's disease compared to the PBO-treated group

**Fan et al. 2011**	Placebo or 40 IU RH-I	Acute	40	Schizophrenia	*N* = 30<br>INS *N* = 15<br>PBO *N* = 15	33.3	50 (8)	13.0 (2.0) years	NR	- ↓ in serum insulin levels after INI administration<br>- No difference in plasma glucose levels observed after INI administration	NR	- HVLT<br>- CPT-IP	- Acute INI did not improve immediate recall total, or delayed recall score on the HVLT in SCZ patients in comparison to the placebo condition<br>- Acute INI did not improve the attention in SCZ patients in comparison to the placebo condition
**Fan et al. 2013**	Placebo or 4 x 40 IU/day RH-I	8 weeks	160	Schizophrenia	N = 45<br>INS *N* = 21<br>PBO *N* = 24	80	INS:<br>49.2 (9.3)<br>PBO:<br>43.8 (9.2)	INS:<br>12.9 (2.3) years<br>PBO:<br>11.4 (2.1) years	NR	NR	Number of side effects reported: 22<br>- Wheezing<br>- Coughing<br>- Trouble breathing<br>- Nasal congestion<br>- Vomiting<br>- Hypersalivation<br>- Nausea<br>- Numbness<br>- Poor concentration<br>- Confusion<br>- Insomnia<br>- Drowsiness	- Digit Span<br>- Verbal Fluency Test<br>- TMT-A<br>- TMT-B<br>- HVLT<br>- CPT-IP	- Eight weeks of INI had no effect on the performance of schizophrenia patients on any cognitive tests in comparison to the PBO condition
**McIntyre et al. 2012**	Placebo or 4 x 40 IU/day RH-I	8 weeks	160	Adults with bipolar I/II disorder	*N* = 62<br>INS *N* = 34<br>PBO *N* = 28	53.23	INS:<br>40.76 (10.15)<br>PBO:<br>39.29 (10.41)	INS:<br>16.61 (3.77) years<br>PBO:<br>16.18 (2.55) years	INS:<br>27.85 (6.21)<br>PBO:<br>29.61 (5.20)	- No difference in blood glucose concentration observed following INI administration	Most commonly reported adverse events:<br>INI:<br>- Intranasal irritation<br>- Anxiety<br>- Nose bleed<br><br>PBO:<br>- Nasal irritation<br>- Increased appetite<br>- Light-headedness	- CVLT-II<br>- PDT<br>- TMT-A<br>- TMT-B<br>- DSST<br>- COWAT<br>- VBM<br>- SILS-Abstract Test<br>- CVMT<br>- NART-R	- INI did not improve the performance of patients with BPD on the CVLT-II, PDT, Trails A, DSST, COWAT, VBM, SILS-abstract, CVMT, NART-R tests in comparison to the PBO condition<br>- INI improved performance of Trails B in patients with BPD in comparison to the PBO condition
**Cha et al. 2017**	Placebo or 4 x 40 IU/day RH-I	4 weeks	160	Treatment resistant major depressive disorder	*N* = 35<br>INS *N* = 19<br>PBO *N* = 16	37.14	INS 47.05 (9.47)<br>PBO: 47.13 (10.67)	INS:<br>High School: 15.79%<br>College: 31.58%<br>Undergraduate: 36.84%<br>Graduate/Professional 15.79%<br>PBO:<br>High School: 18.75%<br>College: 12.50%<br>Undergraduate: 25.00%<br>Graduate/Professional: 43.75%	NR	NR	NR	- CVLT-II<br>- D-KEFS<br>- Verbal FluencyTest<br>- TMT-A<br>- TMT-B<br>- DSST<br>- CFQ	- INI did not improve global neurocognition in patients with major depressive disorder
**Hallschmid et al. 2008**	Placebo or 4 x 40 IU/day Insulin Actrapid	8 weeks	160	Obese men	*N = 30*<br>*INS N = 15*<br>*PBO N = 15*	100	INS:<br>33.47 (1.32)<br>PBO:<br>33.87 (2.23)	NR	INS: 32.35 (1.23)<br>PBO: 33.01 (1.52)	- No difference in serum INS levels and plasma glucose levels after INI treatment	No side effects reported.	- Immediate and Delayed Word List Recall<br>- Wordstem Priming Task<br>- Stroop Test	- INI improved delayed recall of words from the word list compared to patients treated with PBO, effect did not apply as much to emotional/food-related words<br>- Acute and prolonged INI had no effect on the performance on wordstem priming test<br>- Acute and prolonged INI had no effect on selective attention (Stroop test performance)
**Testa et al. 2007**	Placebo or RH-I	24 weeks		Adolescents and adults with type 1 diabetes	*N* = 162<br>Adolescents *N* = 60<br>Adults *N* = 102	51.85	Adolescents: 13.8 (1.4)<br>Adults: 38.2 (10.6)	NR	23.0 (3.2)	NR	NR	- Cognitive Functioning Subscale of Quality of Life Outcome	- INI treatment improved cognitive functioning in adults with type 1 diabetes compared to subcutaneous insulin regimens
**Novak et al. 2014**	Placebo or 1 x 40 IU RH-I	Acute	40	Older adults with type 2 DM	DM *N* = 15<br>HC: *N* = 14	41.38	DM: 62.0 (7.9)<br>HC: 60.1 (9.9)	DM:<br>14.3 (3.8) years<br>HC:<br>17.1 (3.2) years	BMI < 40	NR	The protocol was well tolerated, and there were no serious adverse events. There were no hypoglycemic episodes, nasal irritation,or allergic reactions to insulin.	- BVMT<br>- Verbal Fluency Test	- INI led to better performance on the BVMT compared to the PBO condition, and control subjects performed better than DM subjects<br>- INI improved verbal fluency compared to the PBO group, and control subjects on INS performed better than DM subjects on INS on FAS test
**Novak et al. 2019**	Placebo or 40 IU/day RH-I	4 weeks	40	Parkinson's disease	*N* = 14<br>INS *N* = 8<br>PBO *N* = 6	64.29	63.3 (6.2)	NR	INS:<br>23.9 (4.1)<br>PBO:<br>28.8 (6.5)	- No difference in fasting serum glucose levels after INI administration	No study-related side effects/adverse events reported.	- Verbal Fluencey Test	- INI enhanced performance on the FAS test, while PBO group showed decreased performance
**Zwanenburg et al. 2016**	Placebo or RH-I	24 weeks	Dose varied depending on age (refer to Table 1)	Children with Phelan-McDermid syndrome	*N* = 25	24	6.9 (4.0)	NR	NR	NR	Number of side effects reported: 12<br>-Nosebleeds<br>-Irritation of the nasal area<br><br>(reported in both PBO and INI groups, but with no group breakdown)	- Bayley-III-NL<br>- WPPSI-II-NL	- INI improved cognition and social skills for children older than 3 years old; however, due to a small sample size larger study populations are required to prove the therapeutic effect of INI for children with Phelan-McDermid syndrome
**Rosenbloom et al. 2020**	Placebo, 20 IU GLU	8 weeks	20	Down syndrome	*N* = 12<br>GLU *N* = 6<br>PBO *N* = 6	50	42.7 (1.7)	High school: 75%<br>College: 16.7%<br>Missing: 8.3%	NR	- No difference in serum insulin and glucose levels after INI administration compared to baseline	No side effects reported.	- RBMT<br>- FOME	- INI had no significant effect on learning, immediate recall, delayed recall, memory retention, recognition memory, and retention estimate assessed using FOME<br>- PBO-treated group showed significant improvements in immediate recall on RBMT compared to INI-treated group; on the other hand, INI treated group showed trend toward improved memory retention compared to PBO group

AD: Alzheimer's Disease, ADAS-Cog: Alzheimer's Disease Assessment Scale-Cognition Subscale, ASP-I: Insulin Aspart, Bayley-III-NL: Bayley Scales of Infant and Toddler Development, BNT: Boston Naming Test, BPD: Bipolar Disorder, BVMT: Brief Visuospatial Memory Test-Revised, BVRT: Benton Visual Retention Test, CDR: Clinical Dementia Rating, CVLT-II: California Verbal LearningTest–Second Edition, CVMT: Continuous Visual Memory Test, CFQ: Cognitive Failures Questionnaire, COWAT: Controlled Oral Word Association Test, CPT-IP: Continuous Performance Test-Identical Pairs, D: Vitamin D, Det: Insulin Detemir, DSST: Digit Symbol Substitution Test, DM: Diabetes Mellitus, D-KEFS: Delis–Kaplan Executive Function System, DSRS: Dementia Severity Rating Scale, DSST: Digit Symbol Substitution Test, FAQ: Functional Assessment Questionnaire, FOME: Fluid Object Memory Evaluation, GLU: Glulisine, HC: Healthy Controls, HVLT: Hopkins Verbal Learning Test, INI: Intranasal Insulin, INS: Insulin, MCI: Mild Cognitive Impariment, NART-R: National Adult Reading Test, NR: Not Reported, PBO: Placebo, PDT: Process Dissociation Task, RBANS: Repeatable Battery for the Asessemnt of Neuropsychological Status, RBMT: Rivermead Behavioural Memory Test, RH-I: Regular Human Insulin, SILS-Abstract Test: Shipley Institute of Living Scale (SILS)-Abstract Test, TMT-A: Trail Making Test–Part A, TMT-B: Trail Making Test-Part B, VBM: Visual Backward Masking, WAIS-IV: Wechsler Adult Intelligence Scale - Fourth Edition, WMS: Weschler Memory Scale, WPPSI-II-NL: Wechsler Preschool and Primary Scale of Intelligence

Supplementary Table 3. Neuropsychological tests used to calculate global cognitive score

Cognitive Domains	Cognitive Measures
Working Memory	• Digit Span Test
Verbal Working Memory	• Dot Counting N-back Test
Verbal Memory	• Immediate and Delayed Word List Recall<br>• Immediate and Delayed Story Recall<br>• Delayed Selective Reminding Test Recall<br>• Hopkins Verbal Learning Test<br>• California Verbal Learning Test<br>• Boston Naming Test
Verbal Fluency	• Controlled Oral Word Association Test
Visual Working Memory	• Self-order Pointing Task<br>• Digit Symbol Substitution Test
Visual Learning and Memory	• Continuous Visual Memory Test
Attention	• Rapid Visual Information Processing Task<br>• Visual search<br>• Visual Backward Masking<br>• Continuous Performance Task-Identical Pairs Version
Declarative Memory	• Immediate and Delayed Word List Recall<br>• Immediate and Delayed Story Recall<br>• Word-pair Interference Paradigm<br>• Hopkins Verbal Learning Test<br>• California Verbal Learning Test<br>• Wechsler Memory Scale-IV Logical Memory
Nondeclarative Memory	• Wordstem Priming Task<br>• Finger Sequence Tapping Task
Hippocampal-dependent Memory	• 2D Object Location Task<br>• Process Dissociation Task<br>• Benton Visual Retention Test
Executive Function	• Stroop Test<br>• Mirror Tracing<br>• Trails Making Test A and B<br>• Delis-Kaplan Executive Function System
General Intellectual Ability	• Shipley Institute of Living Scale<br>• National Adult Reading Test
Inhibitory Control	• Eriksen Flanker Task
Dementia	• Alzheimer's Disease Assessment Scale-Cognitive Subscale<br>• Dementia Severity Rating Scale

Supplementary Figure 1. Number of Reports of Nasal Irritation/Rhinitis with INI versus Placebo

Study or Subgroup	Intranasal Insulin Events	Intranasal Insulin Total	Intranasal Placebo Events	Intranasal Placebo Total	Weight	Odds Ratio M–H, Random, 95% CI
Claxton 2015 – 20 IU	1	21	2	20	11.1%	0.45 [0.04, 5.39]
Claxton 2015 – 40 IU	1	19	2	20	11.1%	0.50 [0.04, 6.02]
Craft 2012 – 40 IU	8	38	1	30	14.0%	7.73 [0.91, 65.77]
Craft 2012 –20 IU	4	36	1	30	13.0%	3.63 [0.38, 34.33]
McIntyre 2012	5	34	6	28	26.3%	0.63 [0.17, 2.34]
Svitak 2021 – 40 IU	11	19	5	16	24.5%	3.02 [0.75, 12.21]
Total (95% CI)		167		144	100.0%	1.55 [0.61, 3.91]
Total events	30		17			

Heterogeneity: $Tau^2 = 0.40$; $Chi^2 = 7.21$, df = 5 (P = 0.21); $I^2 = 31\%$
Test for overall effect: Z = 0.93 (P = 0.35)

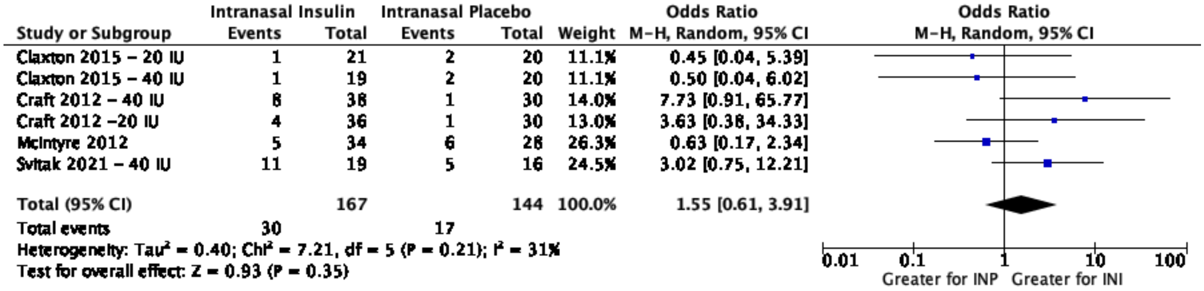

Supplementary Figure 2. Number of Reports of Light-Headedness/Dizziness with INI versus Placebo

Study or Subgroup	Intranasal Insulin Events	Intranasal Insulin Total	Intranasal Placebo Events	Intranasal Placebo Total	Weight	Odds Ratio M–H, Random, 95% CI
Claxton 2015 – 20 IU	1	21	3	20	15.7%	0.28 [0.03, 2.98]
Claxton 2015 – 40 IU	1	19	3	20	15.7%	0.31 [0.03, 3.33]
Craft 2012 – 40 IU	5	38	3	30	37.7%	1.36 [0.30, 6.23]
Craft 2012 –20 IU	3	36	3	30	30.9%	0.82 [0.15, 4.39]
Total (95% CI)		114		100	100.0%	0.72 [0.28, 1.84]
Total events	10		12			

Heterogeneity: $Tau^2 = 0.00$; $Chi^2 = 1.78$, df = 3 (P = 0.62); $I^2 = 0\%$
Test for overall effect: Z = 0.68 (P = 0.50)

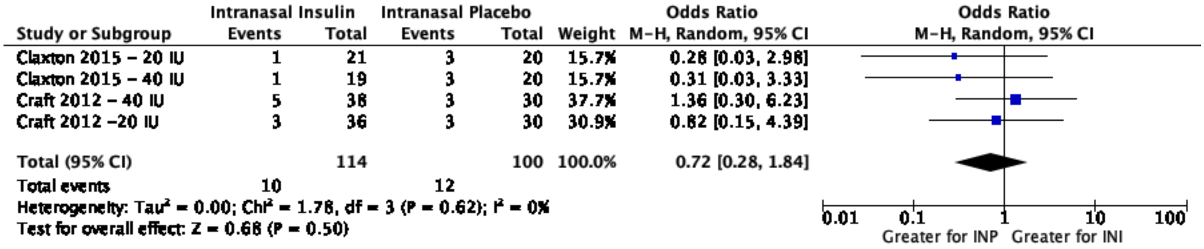

Supplementary Figure 3. Number of Reports of Nausea with INI versus Placebo

Study or Subgroup	Intranasal Insulin Events	Intranasal Insulin Total	Intranasal Placebo Events	Intranasal Placebo Total	Weight	Odds Ratio M–H, Random, 95% CI
Claxton 2015 – 20 IU	0	21	2	20	31.5%	0.17 [0.01, 3.82]
Claxton 2015 – 40 IU	0	19	2	20	31.4%	0.19 [0.01, 4.22]
Svitak 2021 – 40 IU	1	19	1	16	37.1%	0.83 [0.05, 14.48]
Total (95% CI)		59		56	100.0%	0.32 [0.06, 1.81]
Total events	1		5			

Heterogeneity: $Tau^2 = 0.00$; $Chi^2 = 0.70$, df = 2 (P = 0.70); $I^2 = 0\%$
Test for overall effect: Z = 1.29 (P = 0.20)

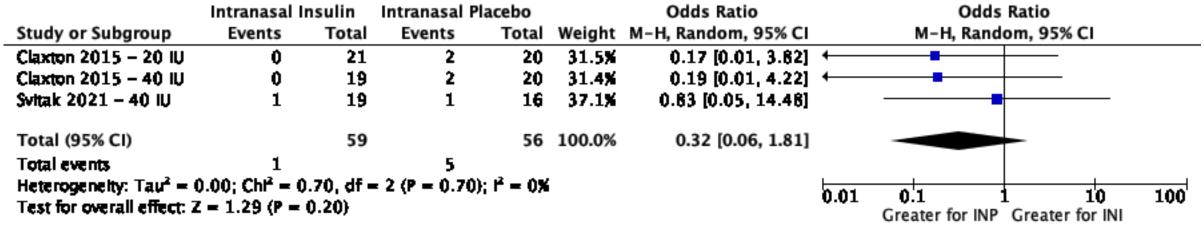

Supplementary Figure 4. Number of Reports of Nose Bleeds with INI versus Placebo

Study or Subgroup	Intranasal Insulin Events	Intranasal Insulin Total	Intranasal Placebo Events	Intranasal Placebo Total	Weight	Odds Ratio M–H, Random, 95% CI
Craft 2012 – 40 IU	3	38	0	30	48.6%	6.01 [0.30, 121.09]
Craft 2012 –20 IU	6	36	0	30	51.4%	13.00 [0.70, 241.01]
Total (95% CI)		74		60	100.0%	8.94 [1.10, 72.50]
Total events	9		0			

Heterogeneity: $Tau^2 = 0.00$; $Chi^2 = 0.13$, df = 1 (P = 0.72); $I^2 = 0\%$
Test for overall effect: Z = 2.05 (P = 0.04)

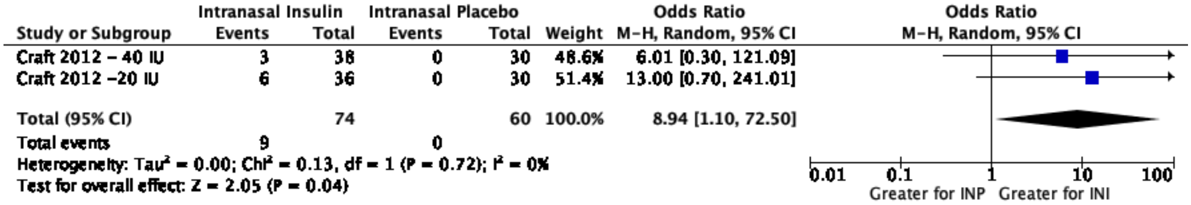

Supplementary Figure 5. Total Number of Adverse Events with INI versus Placebo (Note: for some studies the Odd's ratio is not estimatable as total number of events exceeds sample size for each group; this reflects the fact that some participants may have experienced more than one adverse event).

	Intranasal Insulin		Intranasal Placebo			Odds Ratio
Study or Subgroup	Events	Total	Events	Total	Weight	M-H, Random, 95% CI
Claxton 2015 – 20 IU	6	21	15	20	34.4%	0.13 [0.03, 0.53]
Claxton 2015 – 40 IU	8	19	15	20	34.4%	0.24 [0.06, 0.95]
Craft 2012 – 40 IU	51	38	27	30		Not estimable
Craft 2012 –20 IU	55	36	27	30		Not estimable
Fan 2013	20	21	2	24	31.2%	220.00 [18.50, 2615.69]
Svitak 2021 – 40 IU	44	19	36	16		Not estimable
Total (95% CI)		154		140	100.0%	1.66 [0.04, 74.42]
Total events	184		122			

Heterogeneity: $Tau^2 = 10.47$; $Chi^2 = 31.84$, df = 2 ($P < 0.00001$); $I^2 = 94\%$
Test for overall effect: Z = 0.26 (P = 0.79)

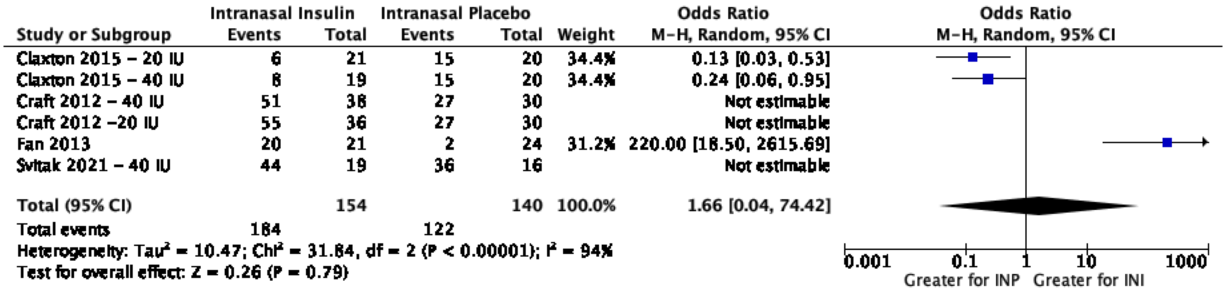

Supplementary Figure 6. Serum Insulin Concentration with INI versus Placebo

Study or Subgroup	Std. Mean Difference	SE	Weight	Std. Mean Difference IV, Random, 95% CI
Benedict 2004	1.01	1.8878	14.0%	1.01 [-2.69, 4.71]
Benedict 2008	0.01	1.0408	26.0%	0.01 [-2.03, 2.05]
Fan 2011	1	1.5919	17.3%	1.00 [-2.12, 4.12]
Hallschmid 2008	-0.42	3.6888	4.9%	-0.42 [-7.65, 6.81]
Novak 2022 – HCs	-2.76	1.5919	17.3%	-2.76 [-5.88, 0.36]
Novak 2022 – T2D	-5.09	1.8521	14.3%	-5.09 [-8.72, -1.46]
Ritze 2018	6.68	14.4952	0.4%	6.68 [-21.73, 35.09]
Rosenbloom 2020 – 20 IU	1.5	3.3419	5.8%	1.50 [-5.05, 8.05]
**Total (95% CI)**			**100.0%**	**-0.80 [-2.50, 0.90]**

Heterogeneity: $Tau^2 = 1.79$; $Chi^2 = 10.42$, df = 7 ($P = 0.17$); $I^2 = 33\%$
Test for overall effect: Z = 0.93 (P = 0.35)

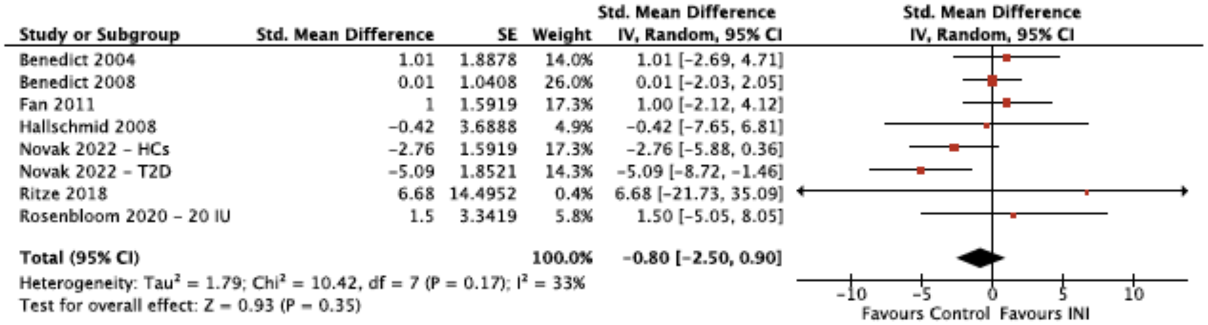

Supplementary Figure 7. Serum Glucose Concentration with INI versus Placebo

Study or Subgroup	Std. Mean Difference	SE	Weight	Std. Mean Difference IV, Random, 95% CI
Benedict 2004	-0.53	0.3316	12.0%	-0.53 [-1.18, 0.12]
Benedict 2008	-0.92	0.2653	15.1%	-0.92 [-1.44, -0.40]
Craft 2020	0.06	0.1378	22.7%	0.06 [-0.21, 0.33]
Fan 2011	-0.07	0.3674	10.6%	-0.07 [-0.79, 0.65]
Hallschmid 2008	0.11	0.3674	10.6%	0.11 [-0.61, 0.83]
Novak 2019	-0.07	0.5408	6.1%	-0.07 [-1.13, 0.99]
Novak 2022 – HCs	-3.86	4.3368	0.1%	-3.86 [-12.36, 4.64]
Novak 2022 – T2D	-0.52	5.0511	0.1%	-0.52 [-10.42, 9.38]
Ritze 2018	0.3	0.2908	13.8%	0.30 [-0.27, 0.87]
Rosenbloom 2020 – 20 IU	-0.29	0.4184	8.9%	-0.29 [-1.11, 0.53]
**Total (95% CI)**			**100.0%**	**-0.18 [-0.48, 0.12]**

Heterogeneity: $Tau^2 = 0.08$; $Chi^2 = 15.75$, df = 9 ($P = 0.07$); $I^2 = 43\%$
Test for overall effect: Z = 1.17 (P = 0.24)

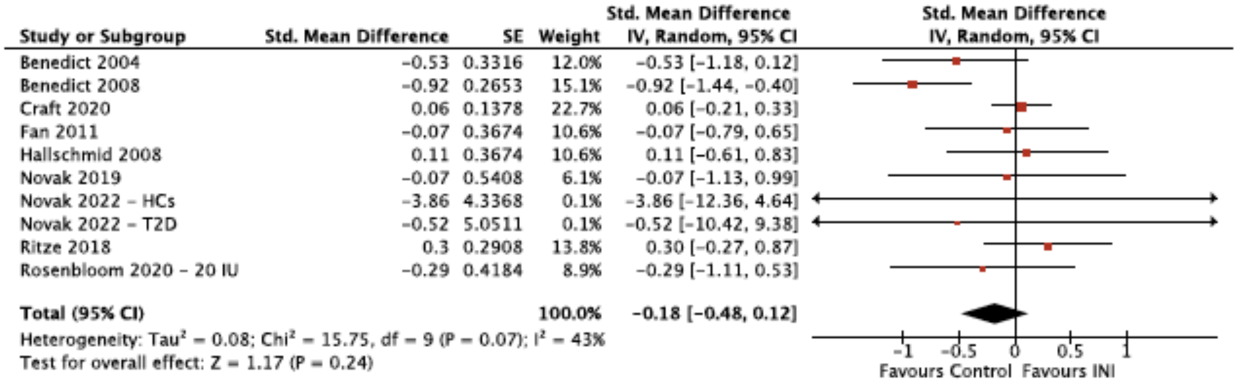

Supplementary Figure 8. Subgroup analysis of age in healthy individuals with INI versus Placebo

Study or Subgroup	Std. Mean Difference	SE	Weight	Std. Mean Difference IV, Random, 95% CI
**65.1.1 Ages 18-35 years**				
Benedict 2004	0.2	0.1378	4.9%	0.20 [−0.07, 0.47]
Benedict 2007 – Insulin Aspart	0.85	0.8368	0.2%	0.85 [−0.79, 2.49]
Benedict 2007 – Regular Human Insulin	0.46	0.4694	0.6%	0.46 [−0.46, 1.38]
Benedict 2008	0.02	0.1225	5.8%	0.02 [−0.22, 0.26]
Feld 2016	−0.1	0.1786	3.3%	−0.10 [−0.45, 0.25]
Gwizdala 2021 – 100 IU	−0.08	0.0816	9.3%	−0.08 [−0.24, 0.08]
Gwizdala 2021 – 120 IU	0.01	0.0816	9.3%	0.01 [−0.15, 0.17]
Gwizdala 2021 – 20 IU	−0.04	0.0969	7.7%	−0.04 [−0.23, 0.15]
Gwizdala 2021 – 40 IU	−0.23	0.1633	3.8%	−0.23 [−0.55, 0.09]
Gwizdala 2021 – 60 IU	−0.06	0.0867	8.7%	−0.06 [−0.23, 0.11]
Gwizdala 2021 – 80 IU	−0.15	0.1122	6.5%	−0.15 [−0.37, 0.07]
Krug 2022	−0.01	0.0663	11.1%	−0.01 [−0.14, 0.12]
Novak 2022	0.78	0.199	2.7%	0.78 [0.39, 1.17]
Ritze 2018	0.24	0.1888	3.0%	0.24 [−0.13, 0.61]
Subtotal (95% CI)			76.7%	0.02 [−0.08, 0.11]
Heterogeneity: Tau² = 0.01; Chi² = 26.79, df = 13 (P = 0.01); I² = 51%				
Test for overall effect: Z = 0.36 (P = 0.72)				
**65.1.2 Ages 36-55 years**				
Hamidovic 2018	0.09	0.1378	4.9%	0.09 [−0.18, 0.36]
Subtotal (95% CI)			4.9%	0.09 [−0.18, 0.36]
Heterogeneity: Not applicable				
Test for overall effect: Z = 0.65 (P = 0.51)				
**65.1.3 Age 55 years+**				
Krug 2010	0.37	0.2704	1.6%	0.37 [−0.16, 0.90]
Novak 2014	0	0.0219	16.9%	0.00 [−0.04, 0.04]
Subtotal (95% CI)			18.5%	0.09 [−0.22, 0.39]
Heterogeneity: Tau² = 0.03; Chi² = 1.86, df = 1 (P = 0.17); I² = 46%				
Test for overall effect: Z = 0.55 (P = 0.58)				
**Total (95% CI)**			100.0%	0.02 [−0.05, 0.09]
Heterogeneity: Tau² = 0.01; Chi² = 29.13, df = 16 (P = 0.02); I² = 45%				
Test for overall effect: Z = 0.45 (P = 0.65)				
Test for subgroup differences: Chi² = 0.39, df = 2 (P = 0.82), I² = 0%				

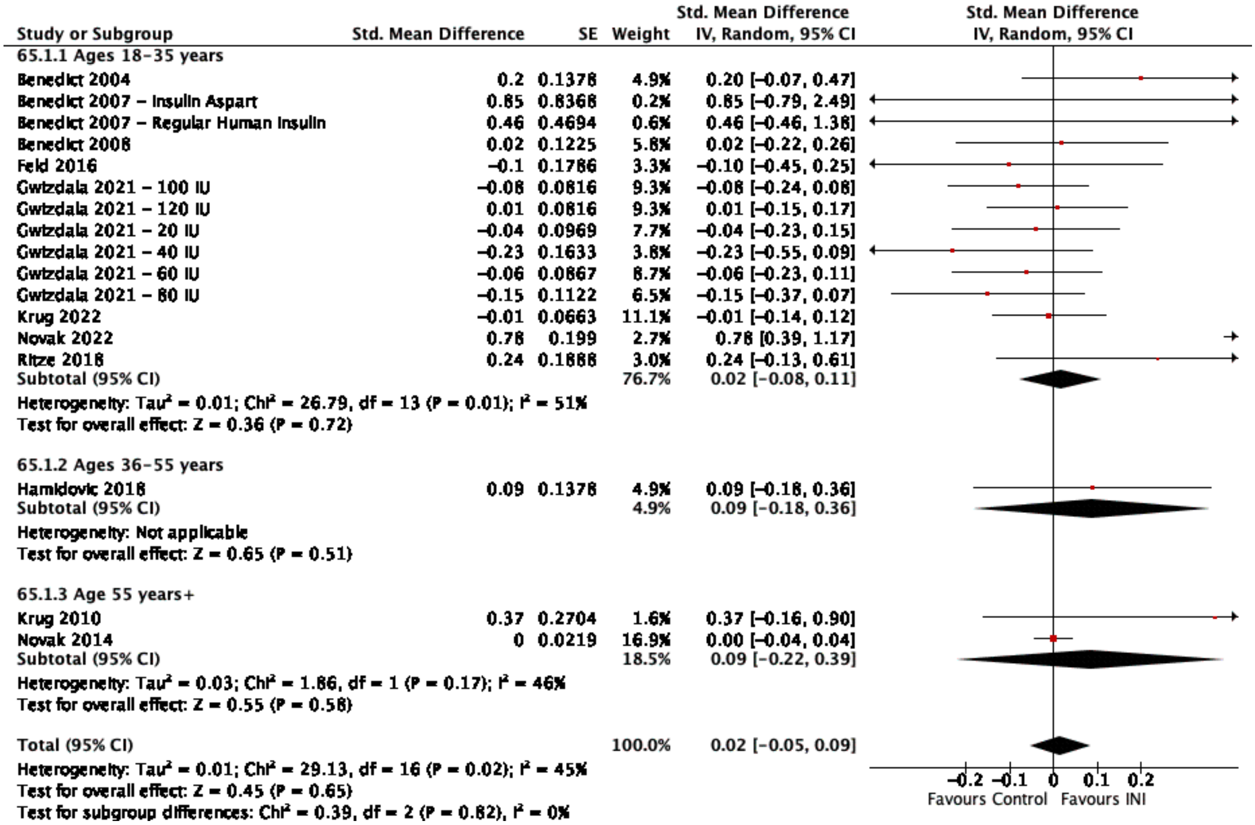

Supplementary Figure 9. Subgroup analysis of sex in healthy individuals with INI versus Placebo

Study or Subgroup	Std. Mean Difference	SE	Weight	Std. Mean Difference IV, Random, 95% CI
**66.1.1 <50% Males**				
Benedict 2008	0.02	0.1225	5.8%	0.02 [−0.22, 0.26]
Gwizdala 2021 – 100 IU	−0.08	0.0816	9.3%	−0.08 [−0.24, 0.08]
Gwizdala 2021 – 120 IU	0.01	0.0816	9.3%	0.01 [−0.15, 0.17]
Gwizdala 2021 – 20 IU	−0.04	0.0969	7.7%	−0.04 [−0.23, 0.15]
Gwizdala 2021 – 40 IU	−0.23	0.1633	3.8%	−0.23 [−0.55, 0.09]
Gwizdala 2021 – 60 IU	−0.06	0.0867	8.7%	−0.06 [−0.23, 0.11]
Gwizdala 2021 – 80 IU	−0.15	0.1122	6.5%	−0.15 [−0.37, 0.07]
Krug 2010	0.37	0.2704	1.6%	0.37 [−0.16, 0.90]
Novak 2014	0	0.0219	16.9%	0.00 [−0.04, 0.04]
Subtotal (95% CI)			69.4%	−0.01 [−0.05, 0.02]
Heterogeneity: Tau² = 0.00; Chi² = 6.81, df = 8 (P = 0.56); I² = 0%				
Test for overall effect: Z = 0.69 (P = 0.49)				
**66.1.2 50% Males**				
Feld 2016	−0.1	0.1786	3.3%	−0.10 [−0.45, 0.25]
Subtotal (95% CI)			3.3%	−0.10 [−0.45, 0.25]
Heterogeneity: Not applicable				
Test for overall effect: Z = 0.56 (P = 0.58)				
**66.1.3 >50% Males**				
Benedict 2004	0.2	0.1378	4.9%	0.20 [−0.07, 0.47]
Benedict 2007 – Insulin Aspart	0.85	0.8368	0.2%	0.85 [−0.79, 2.49]
Benedict 2007 – Regular Human Insulin	0.46	0.4694	0.6%	0.46 [−0.46, 1.38]
Hamidovic 2018	0.09	0.1378	4.9%	0.09 [−0.18, 0.36]
Krug 2022	−0.01	0.0663	11.1%	−0.01 [−0.14, 0.12]
Novak 2022	0.78	0.199	2.7%	0.78 [0.39, 1.17]
Ritze 2018	0.24	0.1888	3.0%	0.24 [−0.13, 0.61]
Subtotal (95% CI)			27.3%	0.24 [0.02, 0.46]
Heterogeneity: Tau² = 0.04; Chi² = 16.87, df = 6 (P = 0.010); I² = 64%				
Test for overall effect: Z = 2.16 (P = 0.03)				
**Total (95% CI)**			100.0%	0.02 [−0.05, 0.09]
Heterogeneity: Tau² = 0.01; Chi² = 29.13, df = 16 (P = 0.02); I² = 45%				
Test for overall effect: Z = 0.45 (P = 0.65)				
Test for subgroup differences: Chi² = 5.31, df = 2 (P = 0.07), I² = 62.3%				

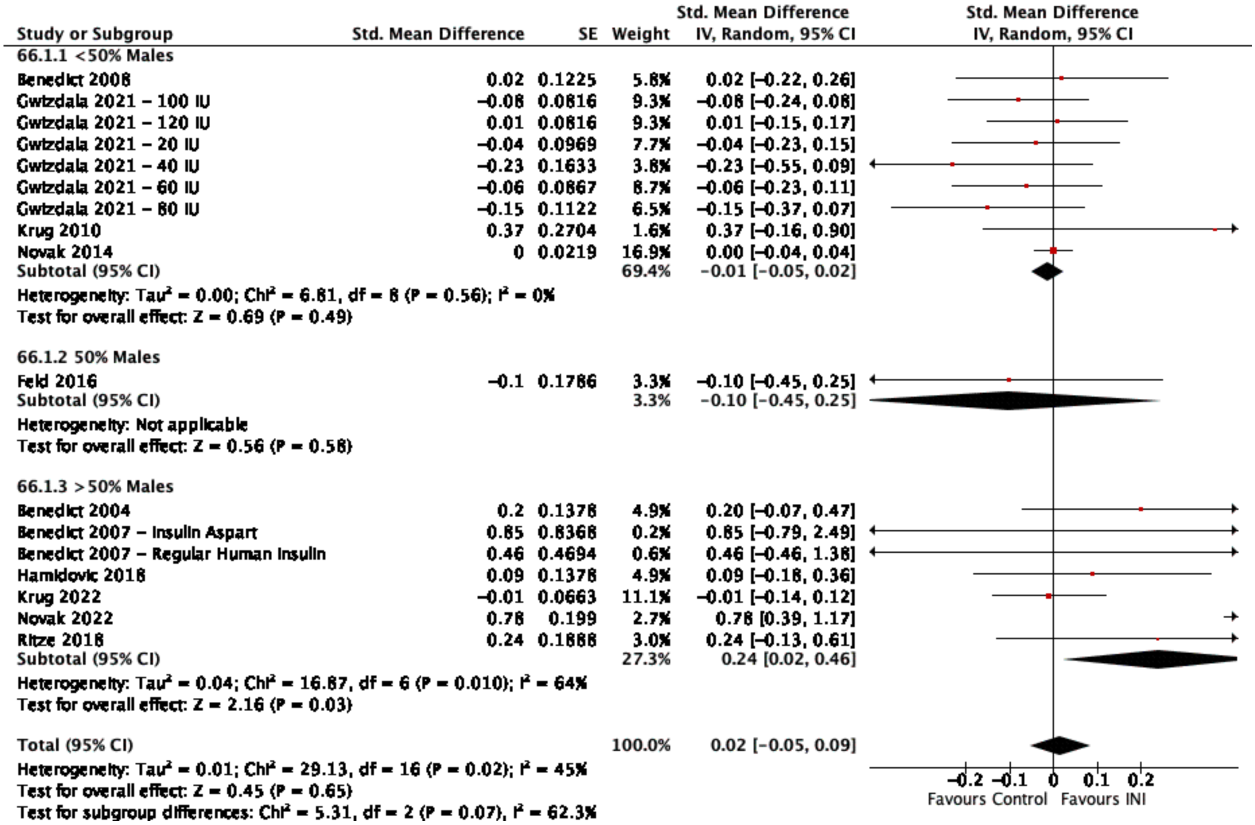

Supplementary Figure 10. Subgroup analysis of sex in patients with AD/MCI with INI versus Placebo

Study or Subgroup	Std. Mean Difference	SE	Weight	Std. Mean Difference IV, Random, 95% CI
**64.1.2 <50% Male**				
Craft 2017	0.71	0.4337	14.7%	0.71 [−0.14, 1.56]
Craft 2017a	0.37	0.4235	14.9%	0.37 [−0.46, 1.20]
Mustapic 2019 − 20 IU	1.32	0.2908	17.5%	1.32 [0.75, 1.89]
Mustapic 2019 − 40 IU	1.65	0.3061	17.2%	1.65 [1.05, 2.25]
Reger 2006 − 20 IU	0.01	0.2806	17.7%	0.01 [−0.54, 0.56]
Reger 2006 − 40 IU	0	0.2755	17.8%	0.00 [−0.54, 0.54]
Reger 2008a − 10 IU	−0.02	0.25	0.0%	−0.02 [−0.51, 0.47]
Reger 2008a − 20 IU	−0.04	0.2449	0.0%	−0.04 [−0.52, 0.44]
Reger 2008a − 40 IU	−0.06	0.25	0.0%	−0.06 [−0.55, 0.43]
Reger 2008a − 60 IU	−0.11	0.2449	0.0%	−0.11 [−0.59, 0.37]
Reger 2008b	1	0.2959	0.0%	1.00 [0.42, 1.58]
Subtotal (95% CI)			100.0%	0.68 [0.07, 1.28]
Heterogeneity: $Tau^2 = 0.46$; $Chi^2 = 27.20$, df = 5 ($P < 0.0001$); $I^2 = 82\%$				
Test for overall effect: Z = 2.19 (P = 0.03)				
**64.1.5 50% Male**				
Rosenbloom 2021	−0.01	0.0918	100.0%	−0.01 [−0.19, 0.17]
Subtotal (95% CI)			100.0%	−0.01 [−0.19, 0.17]
Heterogeneity: Not applicable				
Test for overall effect: Z = 0.11 (P = 0.91)				
**64.1.6 >50% Male**				
Claxton 2013 − 20 IU	0.12	0.1735	10.7%	0.12 [−0.22, 0.46]
Claxton 2013 − 40 IU	0.01	0.1735	10.7%	0.01 [−0.33, 0.35]
Claxton 2015 − 20 IU	0.04	0.102	31.0%	0.04 [−0.16, 0.24]
Claxton 2015 − 40 IU	0.22	0.1888	9.0%	0.22 [−0.15, 0.59]
Craft 2012 − 20 IU	0.01	0.1786	10.1%	0.01 [−0.34, 0.36]
Craft 2012 − 40 IU	0	0.1735	10.7%	0.00 [−0.34, 0.34]
Craft 2020	0.24	0.1378	17.0%	0.24 [−0.03, 0.51]
Rosenbloom 2014	−0.18	0.6327	0.8%	−0.18 [−1.42, 1.06]
Subtotal (95% CI)			100.0%	0.09 [−0.02, 0.20]
Heterogeneity: $Tau^2 = 0.00$; $Chi^2 = 2.79$, df = 7 ($P = 0.90$); $I^2 = 0\%$				
Test for overall effect: Z = 1.52 (P = 0.13)				

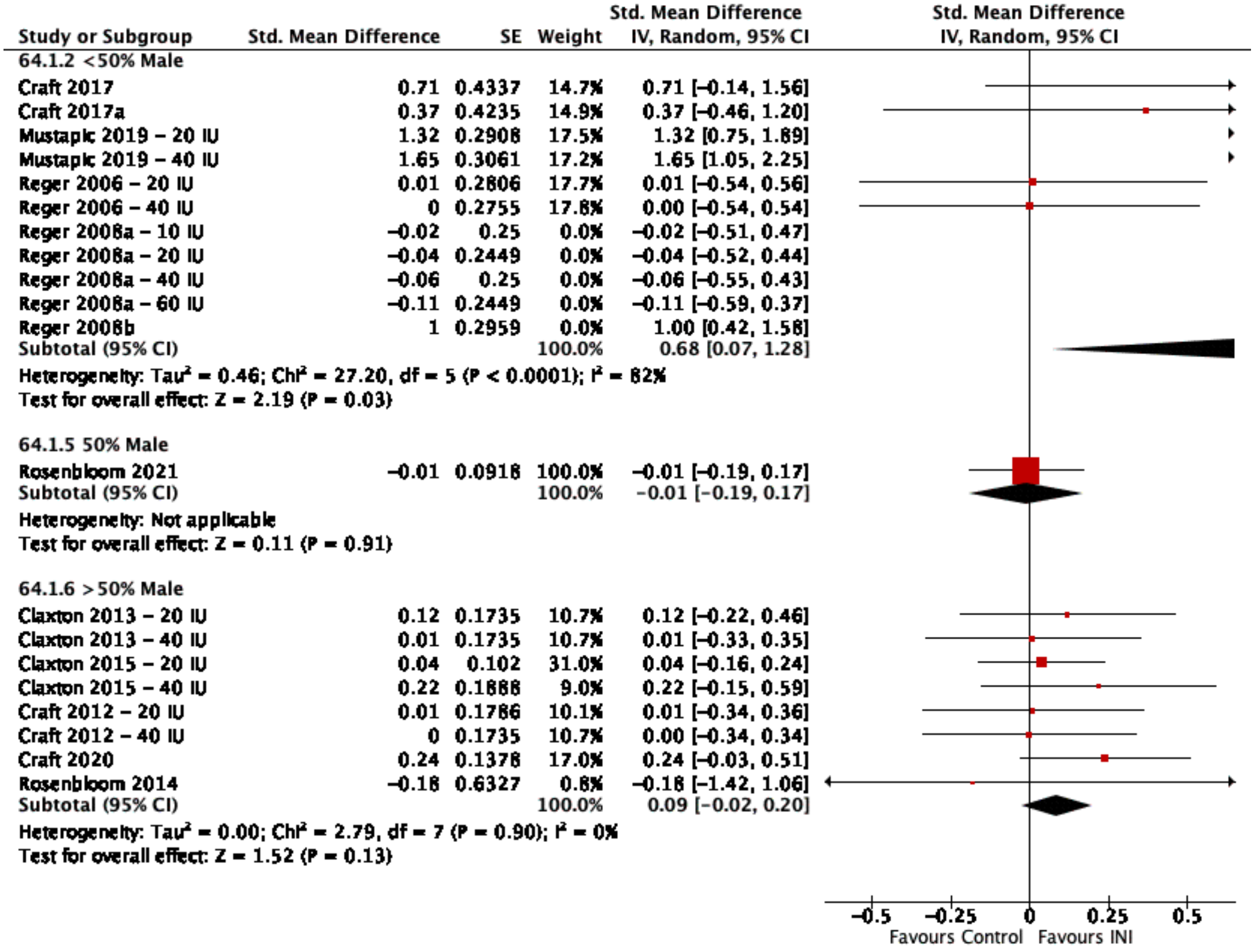

Supplementary Figure 11. Subgroup analysis of dose in patients with AD/MCI with INI versus Placebo

Study or Subgroup	Std. Mean Difference	SE	Weight	Std. Mean Difference IV, Random, 95% CI
**67.1.1 10 IU**				
Reger 2008a – 10 IU	−0.02	0.25	100.0%	−0.02 [−0.51, 0.47]
Subtotal (95% CI)			100.0%	−0.02 [−0.51, 0.47]
Heterogeneity: Not applicable				
Test for overall effect: Z = 0.08 (P = 0.94)				
**67.1.2 20 IU**				
Claxton 2013 – 20 IU	0.12	0.1735	14.7%	0.12 [−0.22, 0.46]
Claxton 2015 – 20 IU	0.04	0.102	19.3%	0.04 [−0.16, 0.24]
Craft 2012 – 20 IU	0.01	0.1786	14.4%	0.01 [−0.34, 0.36]
Mustapic 2019 – 20 IU	1.32	0.2908	8.9%	1.32 [0.75, 1.89]
Reger 2006 – 20 IU	0.01	0.2806	9.3%	0.01 [−0.54, 0.56]
Reger 2008a – 20 IU	−0.04	0.2449	10.6%	−0.04 [−0.52, 0.44]
Rosenbloom 2014	−0.18	0.6327	2.7%	−0.18 [−1.42, 1.06]
Rosenbloom 2021	−0.01	0.0918	19.9%	−0.01 [−0.19, 0.17]
Subtotal (95% CI)			100.0%	0.13 [−0.08, 0.35]
Heterogeneity: $Tau^2 = 0.05$; $Chi^2 = 19.92$, df = 7 (P = 0.006); $I^2 = 65\%$				
Test for overall effect: Z = 1.21 (P = 0.23)				
**67.1.3 40 IU**				
Claxton 2013 – 40 IU	0.01	0.1735	12.2%	0.01 [−0.33, 0.35]
Claxton 2015 – 40 IU	0.22	0.1888	11.6%	0.22 [−0.15, 0.59]
Craft 2012 – 40 IU	0	0.1735	12.2%	0.00 [−0.34, 0.34]
Craft 2017	0.71	0.4337	6.4%	0.71 [−0.14, 1.56]
Craft 2017a	0.37	0.4235	6.6%	0.37 [−0.46, 1.20]
Craft 2020	0.24	0.1378	13.0%	0.24 [−0.03, 0.51]
Mustapic 2019 – 40 IU	1.65	0.3061	8.9%	1.65 [1.05, 2.25]
Reger 2006 – 40 IU	0	0.2755	9.6%	0.00 [−0.54, 0.54]
Reger 2008a – 40 IU	−0.06	0.25	10.3%	−0.06 [−0.55, 0.43]
Reger 2008b	1	0.2959	9.2%	1.00 [0.42, 1.58]
Subtotal (95% CI)			100.0%	0.36 [0.07, 0.65]
Heterogeneity: $Tau^2 = 0.15$; $Chi^2 = 34.93$, df = 9 (P < 0.0001); $I^2 = 74\%$				
Test for overall effect: Z = 2.47 (P = 0.01)				
**67.1.5 60 IU**				
Reger 2008a – 60 IU	−0.11	0.2449	100.0%	−0.11 [−0.59, 0.37]
Subtotal (95% CI)			100.0%	−0.11 [−0.59, 0.37]
Heterogeneity: Not applicable				
Test for overall effect: Z = 0.45 (P = 0.65)				

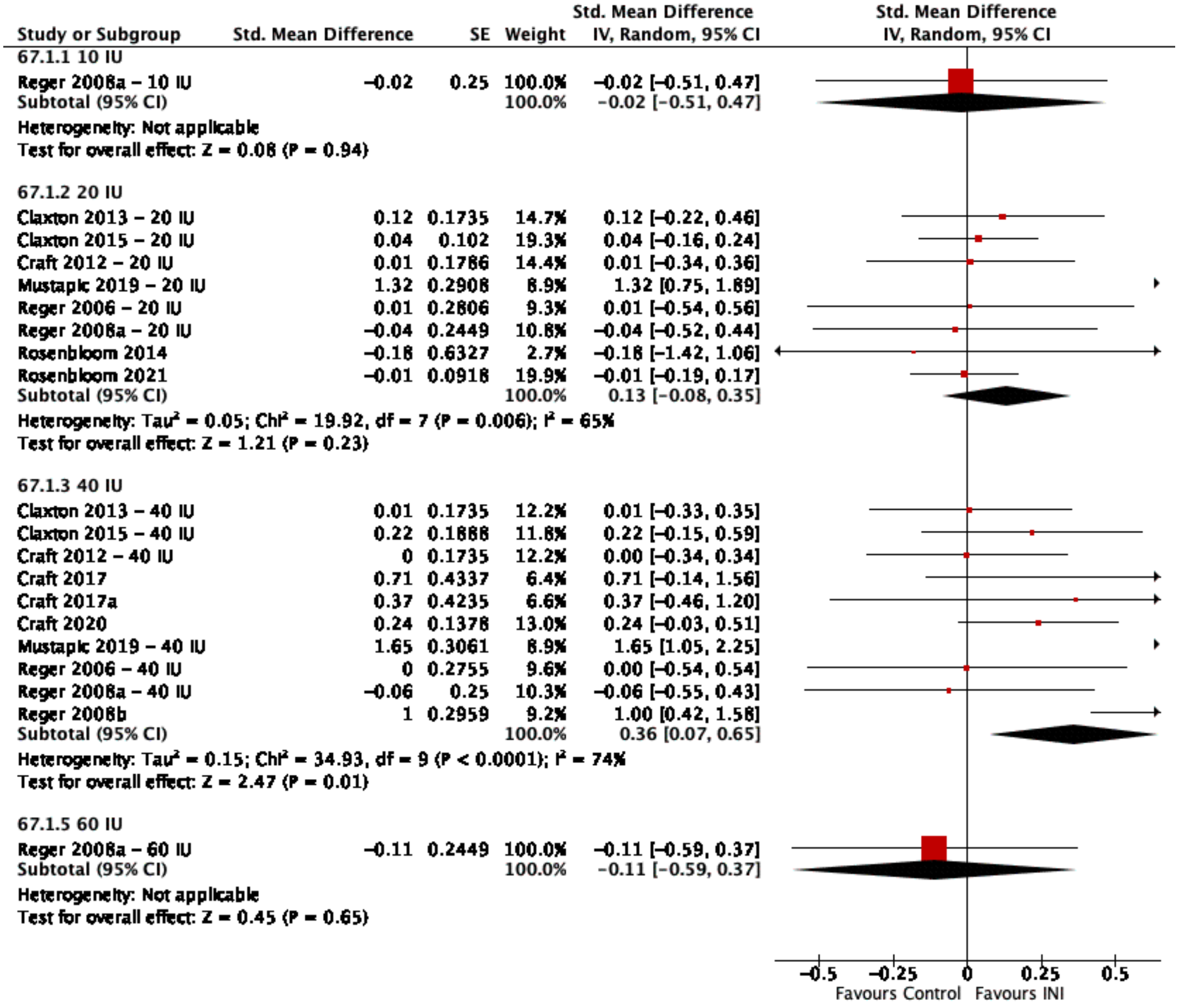

Supplementary Figure 12. Subgroup analysis of duration of exposure (acute versus long-term interventions) across all patient populations

Study or Subgroup	Std. Mean Difference	SE	Weight	Std. Mean Difference IV, Random, 95% CI
**3.2.1 Acute Administration Studies**				
Fan 2011	0.23	0.2092	16.7%	0.23 [−0.18, 0.64]
Novak 2014	0.19	0.2092	16.7%	0.19 [−0.22, 0.60]
Reger 2006 – 20 IU	0.01	0.2806	9.3%	0.01 [−0.54, 0.56]
Reger 2006 – 40 IU	0	0.2755	9.6%	0.00 [−0.54, 0.54]
Reger 2008a – 10 IU	−0.02	0.25	11.7%	−0.02 [−0.51, 0.47]
Reger 2008a – 20 IU	−0.04	0.2449	12.2%	−0.04 [−0.52, 0.44]
Reger 2008a – 40 IU	−0.06	0.25	11.7%	−0.06 [−0.55, 0.43]
Reger 2008a – 60 IU	−0.11	0.2449	12.2%	−0.11 [−0.59, 0.37]
Subtotal (95% CI)			100.0%	0.04 [−0.12, 0.21]
Heterogeneity: $Tau^2 = 0.00$; $Chi^2 = 2.07$, df = 7 (P = 0.96); $I^2 = 0\%$				
Test for overall effect: Z = 0.51 (P = 0.61)				
**3.2.2 Long-term Administration Studies**				
Cha 2017	0.21	0.2398	4.7%	0.21 [−0.26, 0.68]
Claxton 2013 – 20 IU	0.12	0.1735	6.0%	0.12 [−0.22, 0.46]
Claxton 2013 – 40 IU	0.01	0.1735	6.0%	0.01 [−0.33, 0.35]
Claxton 2015 – 20 IU	0.04	0.102	7.4%	0.04 [−0.16, 0.24]
Claxton 2015 – 40 IU	0.22	0.1888	5.6%	0.22 [−0.15, 0.59]
Craft 2012 – 20 IU	0.01	0.1786	5.9%	0.01 [−0.34, 0.36]
Craft 2012 – 40 IU	0	0.1735	6.0%	0.00 [−0.34, 0.34]
Craft 2017	0.71	0.4337	2.3%	0.71 [−0.14, 1.56]
Craft 2017a	0.37	0.4235	2.4%	0.37 [−0.46, 1.20]
Craft 2020	0.24	0.1378	6.7%	0.24 [−0.03, 0.51]
Fan 2013	−0.03	0.1173	7.1%	−0.03 [−0.26, 0.20]
Hallschmid 2008	−0.01	0.6378	1.3%	−0.01 [−1.26, 1.24]
McIntyre 2012	0.13	0.1837	5.7%	0.13 [−0.23, 0.49]
Mustapic 2019 – 20 IU	1.32	0.2908	3.9%	1.32 [0.75, 1.89]
Mustapic 2019 – 40 IU	1.65	0.3061	3.7%	1.65 [1.05, 2.25]
Novak 2019	1.23	0.6072	1.4%	1.23 [0.04, 2.42]
Novak 2022 – T2D	0.2	0.2245	5.0%	0.20 [−0.24, 0.64]
Reger 2008b	1	0.2959	3.8%	1.00 [0.42, 1.58]
Rosenbloom 2014	−0.18	0.6327	1.3%	−0.18 [−1.42, 1.06]
Rosenbloom 2020 – 20 IU	0.06	0.148	6.5%	0.06 [−0.23, 0.35]
Rosenbloom 2021	−0.01	0.0918	7.6%	−0.01 [−0.19, 0.17]
Subtotal (95% CI)			100.0%	0.26 [0.11, 0.41]
Heterogeneity: $Tau^2 = 0.07$; $Chi^2 = 63.44$, df = 20 (P < 0.00001); $I^2 = 68\%$				
Test for overall effect: Z = 3.31 (P = 0.0009)				

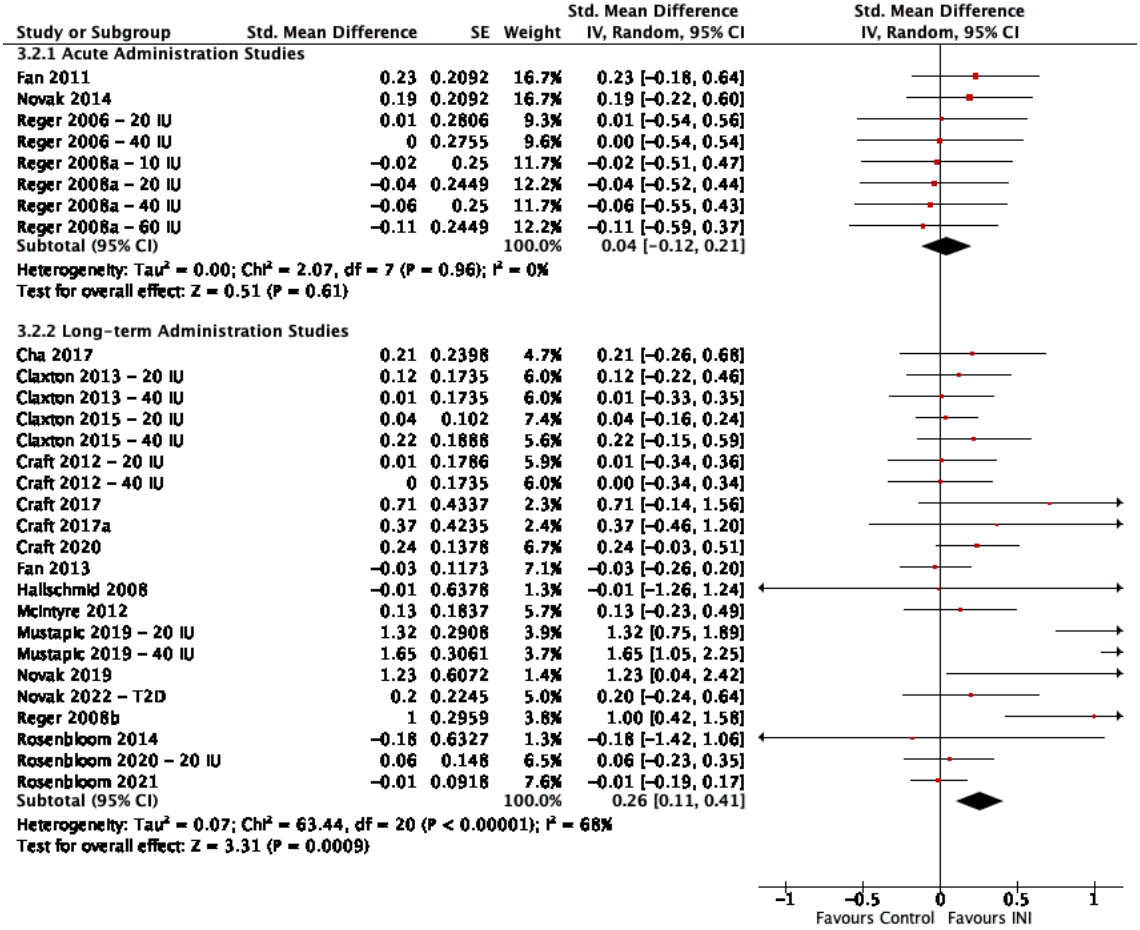

Supplementary Figure 13. Risk of bias graph: review authors' judgements about each risk of bias item presented as percentages across all included studies.

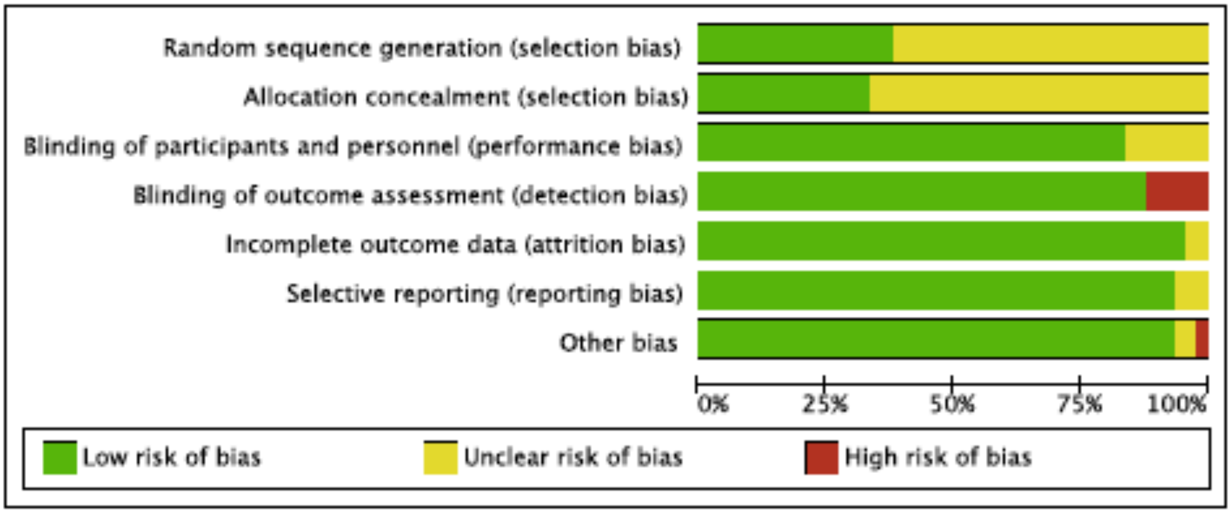

Supplementary Figure 14. Funnel Plot: Assessment of publication bias among healthy control studies.

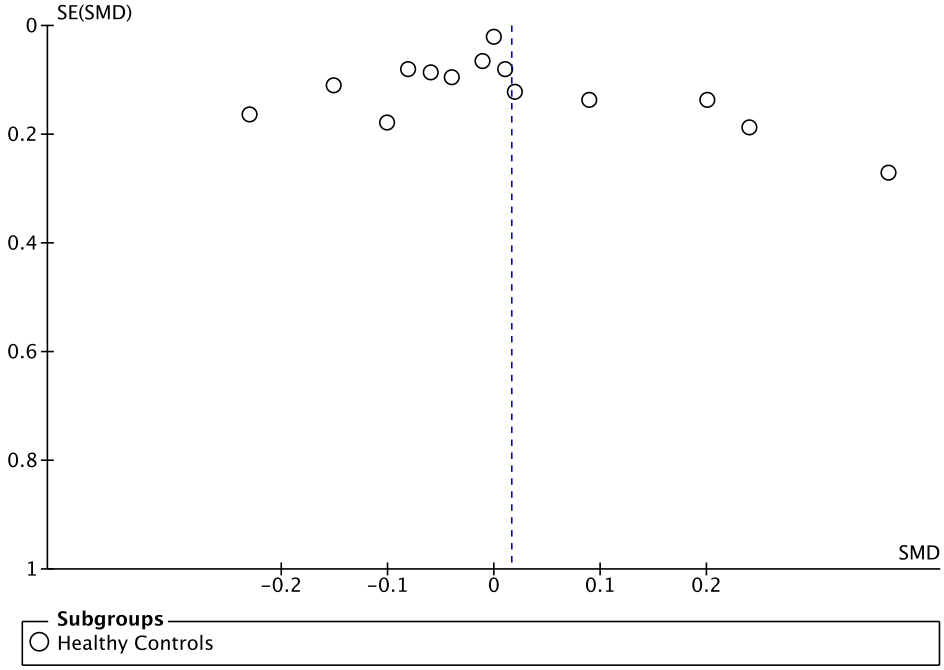

Supplementary Figure 15. Funnel Plot: Assessment of publication bias among patient population studies.

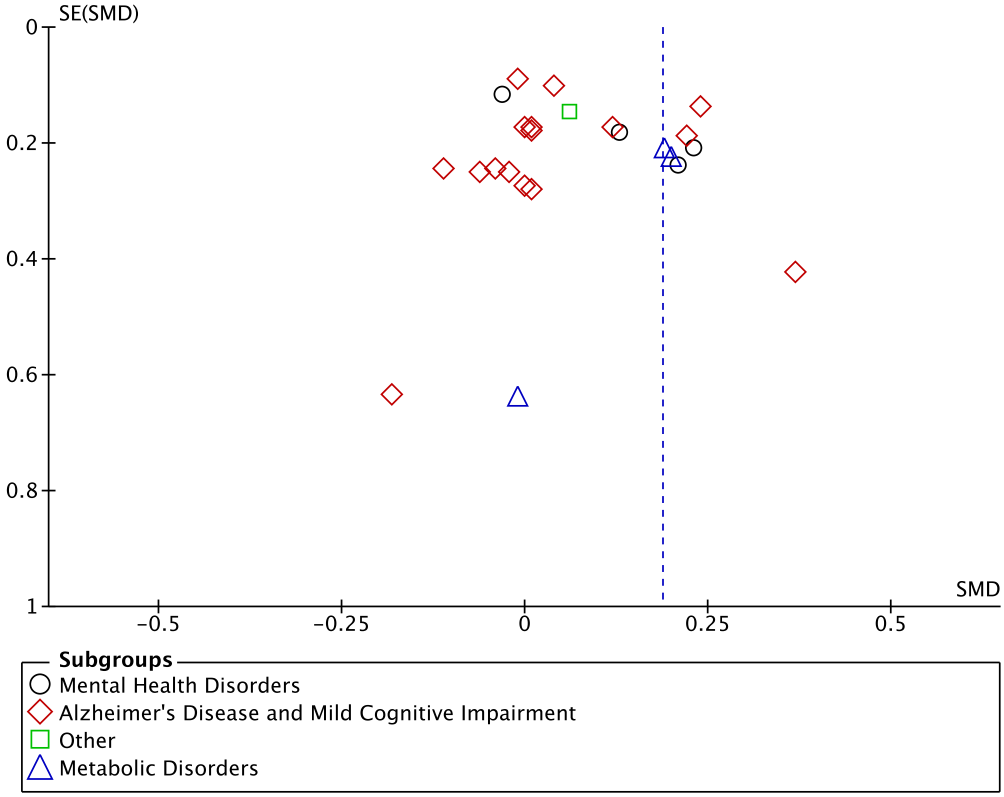

Supplement: S1 File — (PDF) [file pone.0286887.s001.pdf]
