# Supplementary material for: Outcomes and clinical implications of intranasal insulin on cognition in humans: A systematic review and meta-analysis
Source: PLoS One. 2023 Jun 28;18(6):e0286887. doi: 10.1371/journal.pone.0286887 (PMC10306194; doi:10.1371/journal.pone.0286887)
Supplement: Attachment — Submitted filename: Rebuttal Letter.docx [file pone.0286887.s003.docx]

**Rebuttal Letter**

Editor’s Comment

In the case of your submission, we are concerned that the literature review was completed in July 2021. Systematic and scoping reviews should provide an up-to-date overview of the research topic covered. Since the literature search was completed over a year ago, we are concerned that this study does not meet this requirement.

Thank you for this point. We have updated the systematic search as of February 11, 2023. This resulted in the inclusion of three additional studies. The manuscript and analyses have been updated accordingly. We hope this revision will be to the editor’s satisfaction.
